# Oxalate Carbonate Pathway—Conversion and Fixation of Soil Carbon—A Potential Scenario for Sustainability

**DOI:** 10.3389/fpls.2020.591297

**Published:** 2020-12-21

**Authors:** Shameer Syed, Viswanath Buddolla, Bin Lian

**Affiliations:** ^1^College of Life Sciences, Nanjing Normal University, Nanjing, China; ^2^Dr. Buddolla’s Institute of Life Sciences, Tirupati, India

**Keywords:** oxalate/oxalic acid, soil carbon sink, CO_2_ sequestration, carbonates, oxalotrophic bacteria

## Abstract

It is still an important aspect of global climate research to explore a low-cost method that can effectively reduce the increase of CO_2_ concentration in the global atmosphere. Oxalotrophic bacterial communities exist in agricultural or forest soil with ubiquitous oxalate as the only carbon and energy source. When soil oxalate is oxidized and degraded, carbonate is formed along with it. This process is called the oxalate carbonate pathway (OCP), which can increase soil inorganic carbon sink and soil organic matter content. This soil carbon sink is a natural CO_2_ trapping system and an important alternative if it is properly managed for artificial sequestration/storage. As the main driver of OCP, the oxalate degrading bacteria are affected by many factors during the oxalate conversion process. Understanding this process and the synergy of oxalogenic plants, saprophytic decomposers, and oxalotrophic bacteria in agricultural or forest soil is critical to exploiting this natural carbon capture process. This article aims to provide a broader perspective of OCP in CO_2_ sequestration, biomineralization, and elemental cycling.

## Introduction

Carbon dioxide (CO_2_) assimilation by photosynthesis is ubiquitous, whereas mineralization of CO_2_ into inorganic carbon compounds is majorly underrated, which usually involves the synergy of oxalogenic plants, saprophytic decomposers, and oxalotrophic bacteria. The metabolic pathway from oxalate to the mineralization of CO_2_ into carbonates, such as calcium carbonates is “The Oxalate Carbonate Pathway” (OCP) (Braissant et al., 2004; Cailleau et al., 2005; Rowley et al., 2017a; Durand et al., 2018). Mineralized carbon (carbonate) is substantially stable (10^2^–10^6^ years), unlike organic biomass and plays an important role in regulating CO_2_ content in the global C cycle (Cailleau et al., 2011, 2014). Hence, the OCP plays an important role in reducing atmospheric CO_2_ and increasing soil carbon content (Braissant et al., 2004; Cailleau et al., 2005; Rowley et al., 2017a; Durand et al., 2018), but requires numerous, autonomous, biotic, and abiotic components rendering OCP a unique and highly complex phenomenon with limited comprehensive studies (Martin et al., 2012; Cailleau et al., 2014; Rowley et al., 2017b). Despite such tremendous potential of OCP, current research is limited to the mechanism, participating and influencing agents with negligible quantitative characteristics (i.e., quantitative data regarding like net CO_2_ assimilated or carbonate formed is not available in most cases, only qualitative data are presented as OCP is extremely variable). Research on the management and enhancement of net assimilation/sequestration of CO_2_ *via* OCP in different soil environments can have major global implications by impacting net CO_2_ release in major ecosystems like the Amazon rainforest. Furthermore, management/regulation of CO_2_ mineralization *via* OCP influencing CO_2_ net release or assimilation has vast significance in remediating global climatic change. Research into OCP must focus on identifying the beneficial factors or habitat characteristics that result in a net increase in mineralized CO_2_ so that strategies can be used to make maximum use of this phenomenon in large terrestrial ecosystems. In this review, we intend to provide a perspective for the future use and deployment of CaOx generating plants and oxalotrophic bacteria across different scenarios, offering a realistic approach to impacting the natural environment with an outcome including, but not limited to, CO_2_ emissions mitigation, and soil organic carbon (OC) restoration.

## Soil Habitat and OCP-Carbon Sink

Soil is the most complex ecosystem from a biological and geological point of view and is known to be the most vulnerable, prone to various impacts, ranging from erosion to pollution caused partly by nature but mostly due to human activities (Bellard et al., 2012). Furthermore, soil destabilization activities such as deforestation and intensive agricultural practices have reduced soil carbon both above and below ground, resulting in high CO_2_ emissions rather than reduction (Tanveer et al., 2019). The CO_2_ should be sequestrated as stable compounds for the simultaneous reduction of atmospheric CO_2_ concentration and OC depletion (Gross and Harrison, 2019). This can be achieved partly by the microflora involved in oxalate transformations, generating an increase in microcosm density and retaining the carbon sequestrated continuously over a long period, retarding environmental change (Lal, 2004; Lian et al., 2008; Sun et al., 2019a).

### Oxalogenic Plants—Oxalate Bacteria-OCP-Soil Carbon Sink

**FIGURE 1:**
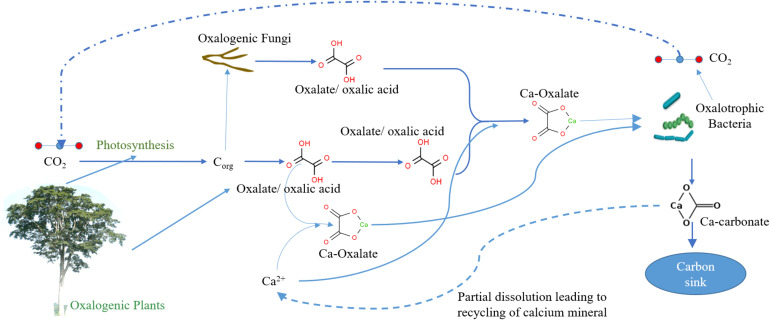
Simplified representation of relationship between plants and fungi (oxalate producers) and oxalotrophic bacteria (oxalate degraders) in creating a soil-based carbon sink.

The biomineralization process involves oxalogenic plants (source of oxalate/oxalic acid) and oxalotrophic bacteria and is very important for certain barren soil ecosystems, particularly in desert soil and Karst topography, since they contain abundant precursors (Lal, 2002; Khaleghi and Rowshanzamir, 2019). Recent studies show many plants species (oxalogenic plants) contain oxalates (of calcium) as part of their metabolism and accumulate in various parts (commonly in roots, leaves, and barks) depending on the type of plant (Figure 1). Oxalogenic plants are imperative for the induction, maintenance, and strengthening of inorganic carbon assimilation (Borrelli et al., 2016; Pierantoni et al., 2018). Furthermore, the termites, saprophytic fungi, and rhizosphere microbial community carry out the degradation of oxalogenic biomass releasing and maintaining the oxalate pool (Cailleau et al., 2011).

The oxalotrophic bacteria (having tolerance to different stresses—pH, moisture content, nutrient availability, contaminants) and oxalogenic crops (Amaranth, Rhubarb, Spinach, etc.) when grown together has the potential to precipitate atmospheric CO_2_ as carbonates (Gadd et al., 2014; Hervé et al., 2018). Oxalotrophic bacterial biomineralization is also beneficial in high calcite soils where OC is limited and rich in mineral calcium which serves as a substrate and is transformed into inorganic carbon (carbonates) (Zhu and Dittrich, 2016). Additionally, the carbonates formed influence the soil’s physico-chemical properties (particularly its pH), which in turn regulate the microbial community and its metabolic activities. Thus, the biomineralization enhances soil modifications that can help crop cultivation (Kalantary and Kahani, 2018; Kurganova et al., 2019; Liu et al., 2019).

### Forest Soil-OCP-Carbon Sink

Forests are the largest reserves of carbon both in the form of vegetation as well as soil OC. The diverse vegetation is crucial to absorb the CO_2_ released by natural and human activities (Zhang et al., 2015, 2018). Atmospheric CO_2_ is assimilated into plants as biomass, much later it accumulates in soil accounting for soil organic matter. Mismanagement of forests leads to deforestation, enhancing CO_2_ emissions during the post-industrial era. CO_2_ concentration in the atmosphere increased drastically to 400 from 270 ppm (pre-industrial era). Forest ecosystems aid in capturing 45% of terrestrial carbon and are responsible for 50% net ecosystem production (Ramachandra and Bharath, 2020). Terrestrial forests play a vital role in the carbon cycle, evident from the sequestration of about 30% of annual global anthropogenic CO_2_ emissions [2 petagrams (Pg) of carbon per year] from the atmosphere (Achat et al., 2015). Thus, soil carbon is the major pool of carbon in terrestrial ecosystem, with a vital role in nutritional security, water quality, biodiversity conservation, and elemental recycling (Chen et al., 2019) (Supplementary Figure S1). The afforestation efforts aimed to increase forest cover should consider the need to develop and sustain biodiversity to mitigate deforestation effects, as observed by some researchers (Qi et al., 2012; Brancalion et al., 2019).

CO_2_ retention in forest soil is dependent on land-use, anthropogenic stress, regimes of disturbance, and prevailing climatic conditions. The natural and intact forests increase the meantime of assimilated carbon’s residence with significantly limited re-emissions (Lal et al., 2015). The Iroko tree (*Milicia excelsa*), a prime example of an oxalogenic tree, produces and stores excess oxalates in its bark and roots, which enter soil over time to be metabolized into carbonates by the oxalotrophic microbiota. This results in the formation of indurated carbonate soils (calcrete forming) in semi-arid soils and affects the cycling of mineral nutrients like calcium, iron, and aluminum. The association and interdependent biomineralization of CO_2_ *via* oxalogenic plants, fungi and oxalotrophic bacteria can therefore be used in monoculture models to establish a healthy terrestrial carbon sink, as proposed by various researchers (Verrecchia et al., 2006; Whiffin et al., 2007; Peng et al., 2008). The oxalotrophic bacteria and oxalogenic trees can be used to cultivate barren landmasses and abandoned agroforestry strips to enhance soil organic content *via* sequestration of soil carbon through OCP. The increase in the forest cover would also mitigate the effect of deforestation and help to stabilize the local and global climate system (Bellassen and Luyssaert, 2014; Borchard et al., 2019).

### Agricultural Soil-OCP-Carbon Sink

Topsoil or the upper layer of the soil is crucial for many of the soil-based operations, such as plant growth, elemental cycling and also serves as a habitat for diverse soil microflora (Ducklow, 2008; Falkowski et al., 2008; Escalas et al., 2019). As previously mentioned, the presence of oxalotrophic bacteria around oxalate-producing plants can cause a pH shift (toward alkaline), promoting the formation of microbial assemblages that further improve carbon assimilation in the soil (Mazen, 2004). This induces OCP, which fixes atmospheric CO_2_ in the form of carbonates. The inorganic component is made of readily available metal in the soil either accumulated from mineral weathering or plant exudates/released from the decay of plant debris and ectomycorrhizal assemblages (mostly calcium) (Aragno and Verrecchia, 2012; Qin et al., 2013). The downside of this phenomenon is the need for the proximal coexistence of oxalogenic plants or oxalate minerals and oxalate-dependent heterotrophic microbiota that use the oxalate and acidic conditions in the habitat (oxalotrophy), leading to OCP (Dauer and Perakis, 2013; Lal et al., 2015). This is particularly effective in soils affected by mining, inorganic contaminants, and low OC content (Karst and sandy soils). With the increase in OC, the physical properties of the soil change dramatically due to the existence of a microbial population and thus affect crucial properties such as water-holding ability and pH (Ye et al., 2018).

These findings can lead to arid, dry, low fertile agroforestry strips being used to create green or agroforestry zones, creating soil-based carbon sinks specifically in areas without previous plant coverage (Supplementary Figure S2). These green zones would fix CO_2_ into plant biomass and create an opening for microbial community establishment. Besides, if the microbial community is controlled by human involvement, the organic biomass can be effectively mineralized, and added to the soil carbon sink.

## Oxalate Carbonate Pathway—Environmental Sustainability

### OCP-Carbon Sink-Soil Stability

The primary cause of soil instability is loose compaction of soil particles and the minerals with which it is made up of Pankova and Gerasimova (2012). The instability of soil leads to the loss of water holding capacity subsequently increasing soil aridity, the foremost reason for desertification. Moreover, desertification decreases biomass generation *via* plant photosynthesis, a major threat to grasslands and agricultural lands throughout the world. To fight desertification, many activities and methods are being utilized, yet the most effective approach, i.e., stabilizing soil particles, thus prevent desertification is left neglected (Cheng et al., 2014, 2017). The precipitation of CO_2_ through OCP by oxalogenic plants and oxalotrophic bacterial populations is efficient in binding the sand grains together, increasing the stability of the topsoil and also creates a carbon sink through carbonate accumulation (Mujah et al., 2016; Jiang et al., 2019; Seifan and Berenjian, 2019). In our laboratory experiments, when Streptomyces NJ10, an oxalotrophic bacteria with exceptional oxalate metabolizing potential isolated from bacterial assemblages in the ectomycorrhizosphere (Sun et al., 2019a), was grown in the presence of oxalate mineral in soils, cementing bridges were formed between the soil particles increasing the compaction of the soil (Supplementary Figure S3). Therefore, it is deduced that induction of CaCO_3_ precipitation binds sand grains together at the particle–particle contacts, increasing soil stability, particularly in loose soils with limited or low OC (DeJong et al., 2010; Mortensen et al., 2011; Chu et al., 2012). Many researchers have reported improvement of soil fertility and OC content when microbial communities capable of enhancing the mineral weathering are established, the CO_3_^2–^ ions precipitate with Ca^2+^ as calcite crystal, which generates cementing bridges between soil particles (Frankel, 2003; Dhami et al., 2013).

### OCP-Heavy Metal Immobilization

Heavy metals like lead (Pb), mercury (Hg), arsenic (As), cadmium (Cd), chromium (Cr, hexavalent), aluminum (Al), etc., are increasingly becoming a severe threat to the environment and the resident biota (Akoto et al., 2018; Al Osman et al., 2019; Obiora et al., 2019). The major sources of heavy metal pollution are inorganic fertilizers, metal-based pesticides and insecticides, industrial effluents, mine, and dumping yard leachate. Among many routes to stop and remove heavy metal contamination, microbial-based remediation techniques are preferential as they are effective, economical, and ecofriendly in their action (Jáuregui-Zúñiga et al., 2005; Pongrac et al., 2018; Tamayo-Figueroa et al., 2019). The oxalotrophic bacteria have great potential in this regard, as they can metabolize oxalic acid and produce carbonates (Aragno and Verrecchia, 2012; Xu et al., 2014; Pongrac et al., 2018), a stable end product, thus modifying the habitat pH and affecting the solubility and mobility of the heavy metals and prevent seeping into the subsoil (as the majority of the metals are insoluble in alkaline pH) (Zhang et al., 2020). Application of oxalic acid-producing heterotrophic fungi like *Aspergillus* spp. along with the oxalotrophic bacteria would possibly be a more effective way to precipitate the heavy metals as carbonates, neutralizing the heavy metal toxicity (Anbu et al., 2016). It is observed in our research when *Aspergillus niger* is grown in the presence of heavy metal Lead, it produced lead oxalate (Unpublished data). Additionally, *Streptomyces* NJ10 an oxalotrophic bacteria when grown in the presence of lead oxalate was able to utilize lead oxalate and grew well. Based on these findings, it can be concluded that heterotrophs capable of producing organic acid and oxalotrophic bacteria may be converted into a microbial preparation and applied to polluted sites to reduce the toxicity of heavy metals.

## Soil Carbon Sink-Factors in Play

**FIGURE 2:**
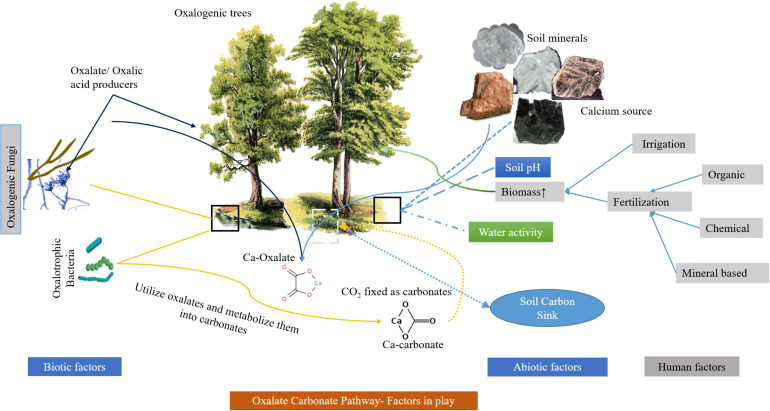
A typical oxalate carbonate pathway showing different biotic and abiotic influencing factors.

Soil carbon sink is suitable for many CO_2_ capture and storage routes but has much slower CO_2_ assimilation rates. The assimilation of CO_2_ in the soil depends on many factors including pH, microbial assemblies, water movement, soil geology, and seasonal weather variability. It is also well established that the microcosms created by ectomycorrhiza of many higher plants support diverse oxalotrophic bacteria and provide a stable oxalate pool along with a more suitable environment for the oxalate mineralization (Guggiari et al., 2011; Sun et al., 2019a). Besides, carbon capture, storage and stability in the form of inorganic compounds is typically dependent on microbial assemblies, plant diversity and geological characteristics in the soil and can be enhanced/influenced by human interventions such as the introduction of organic mineral fertilizers (Lian et al., 2008; Liu et al., 2012; Sun et al., 2019b) (Figure 2). The addition of nitrogen to the soil in the form of bio-organic fertilizer also increases CO_2_ assimilation in the form of OC by the growth of plant biomass as observed in several studies that can be adapted in the agricultural sector (Yang et al., 2020). However, this lacks stability and is promptly moved to the global carbon cycle as CO_2_ within a limited time (approximately 10^1^–10^3^ years). In comparison, mineralized carbon takes longer periods but is greatly influenced by soil pH, since acidic pH dissolves the mineralized carbon (HCO_3_^-^). Some researchers have demonstrated that silicate-rich clay mineral soils are significantly better choices for carbon sinks as they can provide the inorganic (metal) component for carbon mineralization through soil biological activity (Chen et al., 2017; Xiao et al., 2017). Furthermore, deeper studies are required on factors that have a significant impact on the soil carbon sink but have received little attention, including soil carbon management.

## Conclusion and Prospects

The carbon cycle has been extensively studied concerning climate change research and the sequestration of carbon is mostly attributed to OC storage alone in the global carbon cycle. Soil-mineral carbon sinks are of great interest for two reasons: (1) the stability of mineralized carbon (10^2^–10^6^ years) is up to 100,000 times longer than for soil organic matter carbon (10^1^–10^3^ years) and (2) thousands of plant species are known to mineralize atmospheric carbon but are usually ignored, whereas sequestration of carbon in soil may only be considered as OC sink. However, it represents a potentially more effective carbon sink if contained in large amounts, due to the resilience of mineralized carbon in soils. Oxalate and its transformations in various oxalogenic plants can affect the carbon sink of soil and global carbon beyond what was previously understood. Measures such as the conservation of carbon-mineralizing trees such as Iroko (*M. excelsa*) and other biomineralizing plant species in depleted or low-carbon soils are potentially important for significant mineral carbon sinks development. Research on the quantitative assimilation of CO_2_ into mineralized carbon is therefore required to create a commercially viable soil carbon sink, which is practically accessible *via* OCP. And hence, the cultivation of carbon-mineralizing trees in the form of agroforestry projects can serve multiple purposes of carbon assimilation and soil recovery. Finally, the major factor required to resolve sustainable mineralization of carbon is its potential disintegration by metabolic activities of soil bacteria and modifications of soil pH.

## Author Contributions

SS curated related data and prepared the original draft. BL conceptualized and reviewed the draft manuscript. VB reviewed and edited the draft manuscript. All authors contributed to the article and approved the submitted version.

## Conflict of Interest

The authors declare that the research was conducted in the absence of any commercial or financial relationships that could be construed as a potential conflict of interest.

## Supplementary Material

Supplementary Figure 1 Carbon cycling in terrestrial forest ecosystems and its major services.

Supplementary Figure 2 A possible model for utilizing oxalogenic crops/agroforestry and oxalotrophic bacteria to create soil-based carbon sinks in low fertile soils. Inset: typical carbonate mineralization.

Supplementary Figure 3 Stereomicroscope images showing carbonate bridges formed by oxalotrophic bacteria *Streptomyces* NJ10, holding soil particles. **(a)** Carbonate bridge in between two sand particles indicated by arrow. **(b)** similar sand particles lacking such bridges are more prone to erosion (indicated by arrows).
