# Supplementary figures and images for: Oxalate Carbonate Pathway—Conversion and Fixation of Soil Carbon—A Potential Scenario for Sustainability
Source: Front Plant Sci. 2020 Dec 21;11:591297. doi: 10.3389/fpls.2020.591297 (PMC7793669; doi:10.3389/fpls.2020.591297)

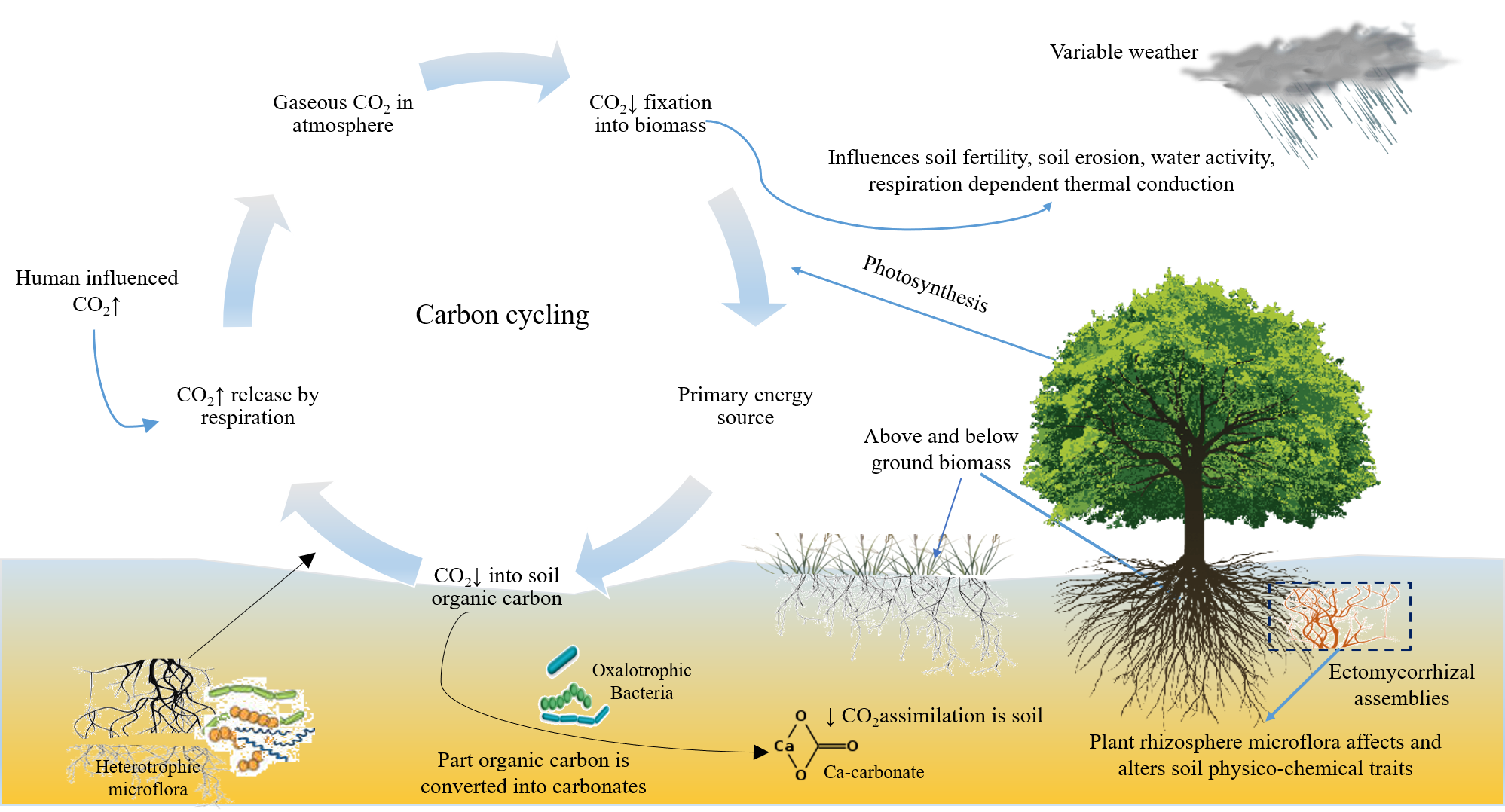

Supplement: Supplementary Figure 1 — Carbon cycling in terrestrial forest ecosystems and its major services. [file Image_1.TIF]

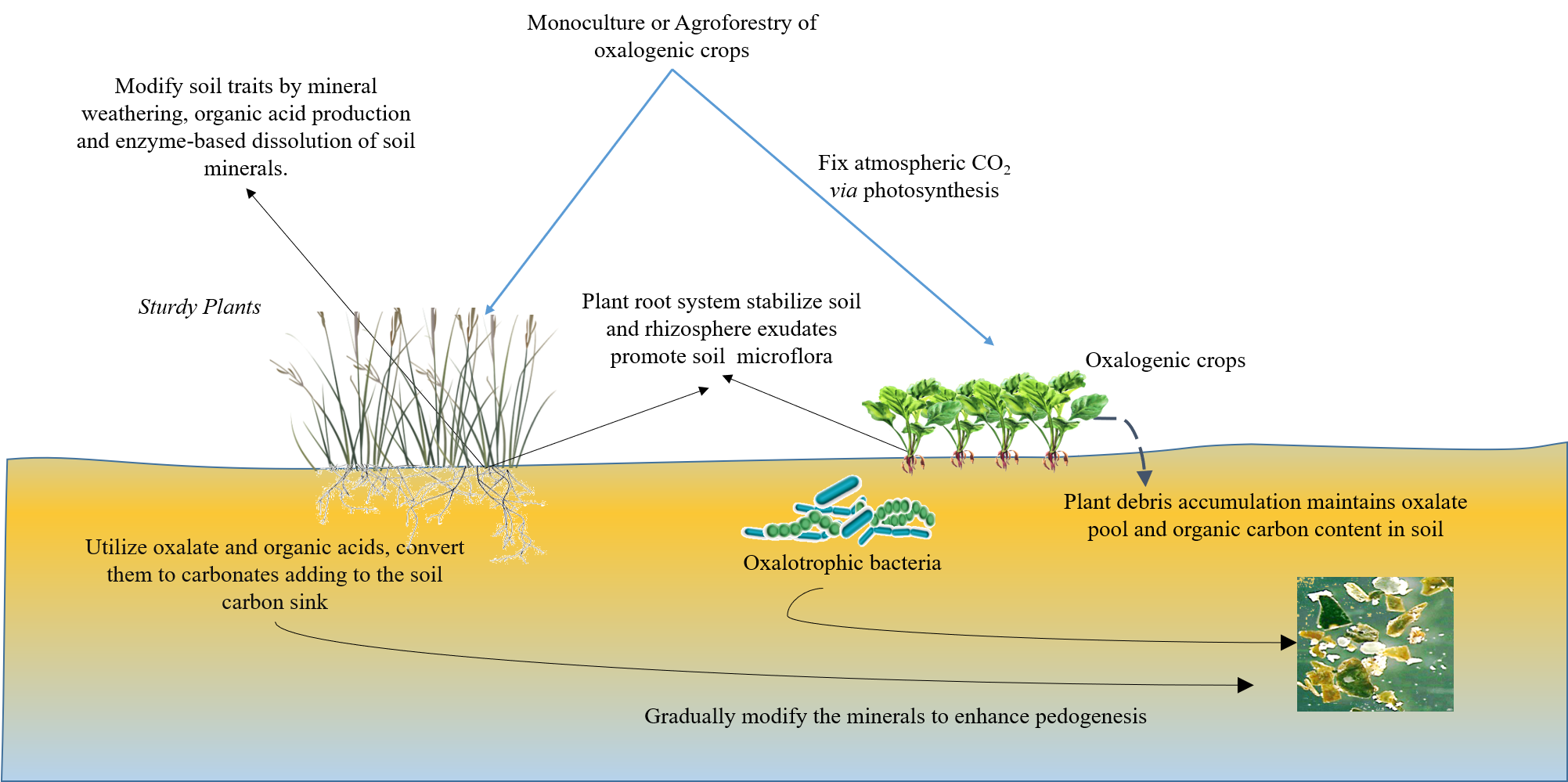

Supplement: Supplementary Figure 2 — A possible model for utilizing oxalogenic crops/agroforestry and oxalotrophic bacteria to create soil-based carbon sinks in low fertile soils. Inset: typical carbonate mineralization. [file Image_2.TIF]

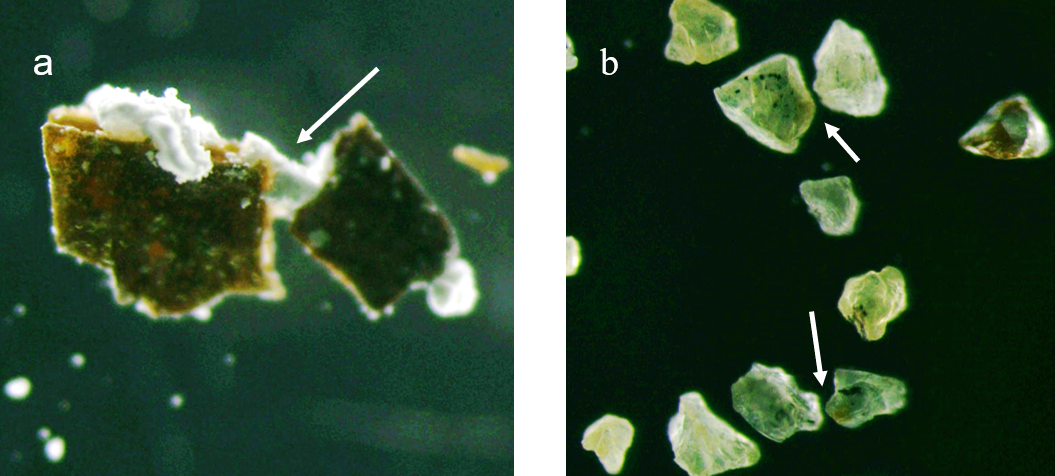

Supplement: Supplementary Figure 3 — Stereomicroscope images showing carbonate bridges formed by oxalotrophic bacteria Streptomyces NJ10, holding soil particles. (a) Carbonate bridge in between two sand particles indicated by arrow. (b) similar sand particles lacking such bridges are more prone to erosion (indicated by arrows). [file Image_3.TIF]
